# Supplementary material for: Bayesian refinement of protein structures and ensembles against SAXS data using molecular dynamics
Source: PLoS Comput Biol. 2017 Oct 18;13(10):e1005800. doi: 10.1371/journal.pcbi.1005800 (PMC5662244; doi:10.1371/journal.pcbi.1005800)
Supplement: S1 Archive — (BZ2) [file pcbi.1005800.s011.tar.bz2 › gromacs_4.62_bayesian_saxs_refi/share/html/online/ndx.html]

ndx file format


## ndx file format

|  |  |
| --- | --- |
| Main Table of Contents | **VERSION 4.6** |
| GROMACS homepage | **Sat 19 Jan 2013** |

---

### Description

The GROMACS index file (usually called index.ndx) contains some
user definable sets of atoms. The file can be read by
most analysis programs, by the graphics program (ngmx)
and by the preprocessor (grompp).
Most of these programs create default index groups when no index
file is supplied, so you only need to make an index file when you need special
groups.

First the group name is written between square brackets.
The following atom numbers may be spread out over as many lines as you like.
The atom numbering starts at 1.

An example file is here:

```
[ Oxygen ]
1 	4 	7 
[ Hydrogen ]
2	3	5	6
8	9
```

There are two groups, and total nine atoms. The first group
**Oxygen** has 3 elements.
The second group **Hydrogen** has 6 elements.

An index file generation tool is available: make\_ndx.

---

http://www.gromacs.org
